# Supplementary material for: Gut microbiome modulates Drosophila aggression through octopamine signaling
Source: Nat Commun. 2021 May 11;12:2698. doi: 10.1038/s41467-021-23041-y (PMC8113466; doi:10.1038/s41467-021-23041-y)
Supplement: Supplementary file 1 — Supplementary Information [file 41467_2021_23041_MOESM1_ESM.pdf]

Gut Microbiome Modulates *Drosophila* Aggression through Octopamine Signaling

Jia et al.

Supplementary Information

Supplementary Figures and Legends

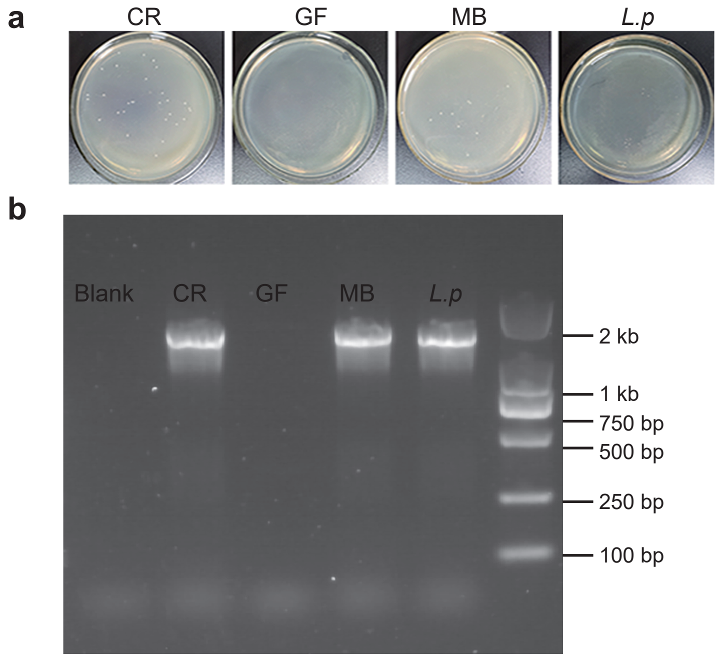

Supplementary Fig. 1 Verification of axenia of GF flies and bacterial re-colonization. (**a**) Bacterial load was determined by plating the homogenate of fly midguts with 1: 1,000 dilution on LB agar plates. Representative images are shown. (**b**) Representative images of DNA agarose electrophoresis using 16S rDNA PCR samples. Experiments were repeated for 3 times. Blank, negative control without DNA templates for PCR reaction; CR, conventionally reared fly; GF, germ free fly; MB, GF fly adding mixed bacterial; *L.p*, GF fly associated with *L.p*.

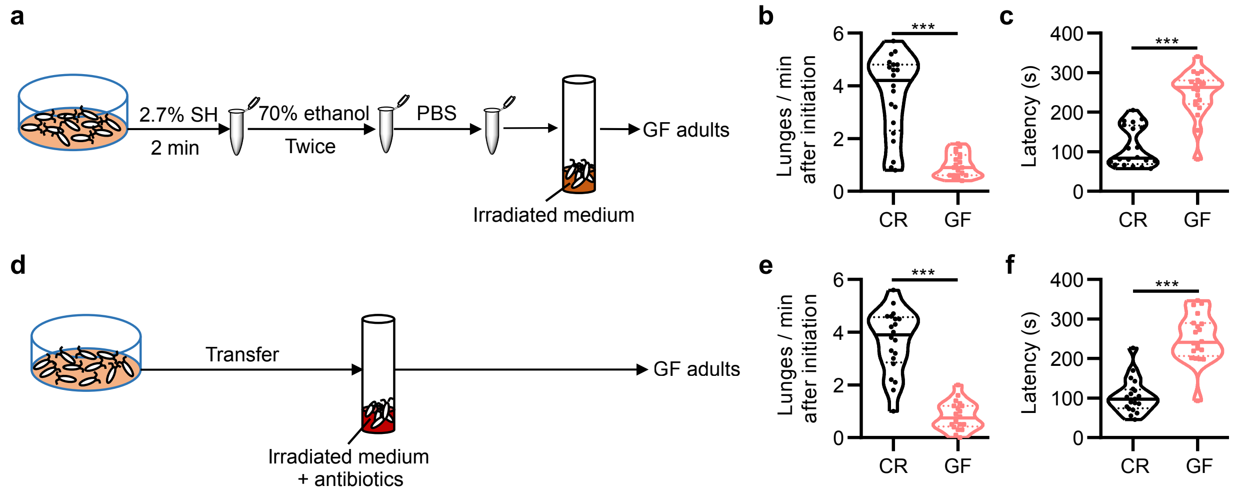

Supplementary Fig. 2 Aggression is reduced in GF flies generated using two additional methods. (**a**) Protocol to generate GF flies using irradiated medium. (**b** and **c**) Lunging frequency (**b**) and latency (**c**) in the above generated GF males were not significantly different from CR males. *n* = 20 for each. (**d**) Protocol to generate GF flies using irradiated medium and antibiotics. (**e** and **f**) Lunging frequency (**e**) and latency (**f**) in the above generated GF males were not significantly different from CR males. *n* = 20 for each. ***$p < 0.001$. Kruskal-Wallis test followed by Dunn's multiple comparisons test.

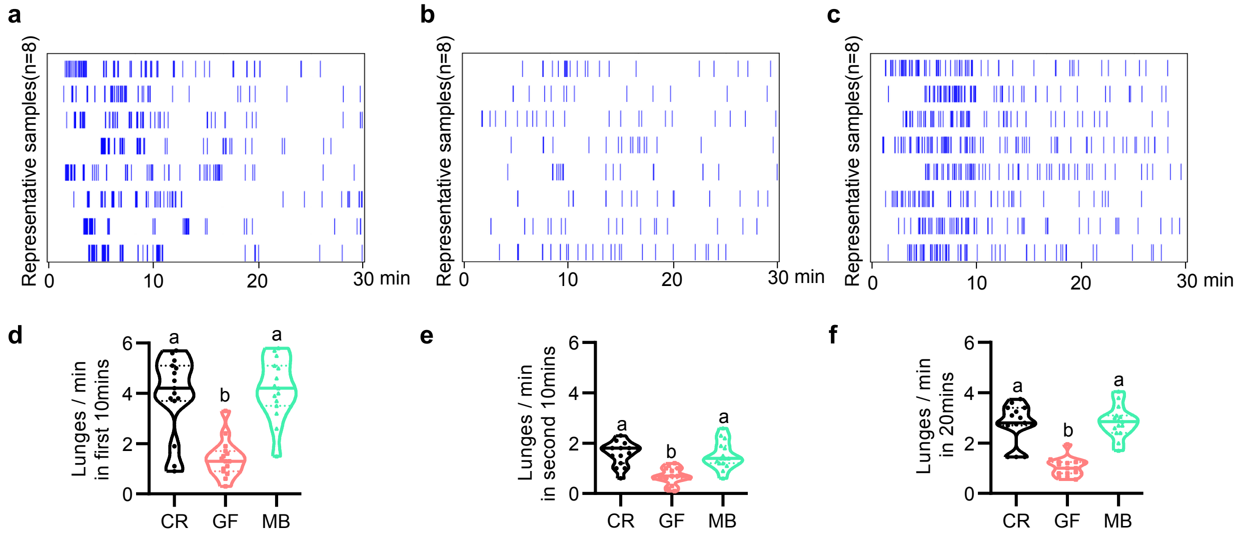

Supplementary Fig. 3 Overview of lunging behavior in the 30-min test. (**a-c**) Raster plots of lunging (blue ticks) in CR (**a**), GF (**b**) and MB (**c**) flies in 30 mins. (**d-f**) Lunging frequencies within first 10 mins (**d**), second 10 mins (**e**) and total 20 mins (**f**) after fighting initiation. $n = 15$ for each. For all variables have different letters, they are significantly different ($p < 0.05$). Kruskal-Wallis test followed by Dunn's multiple comparisons test.

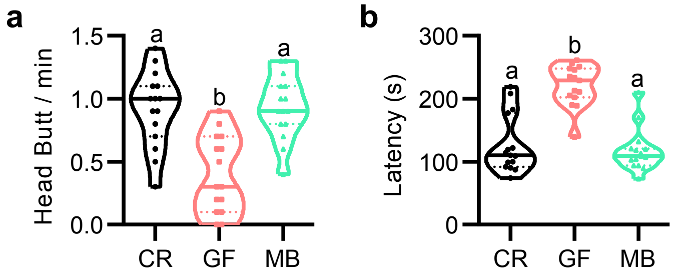

Supplementary Fig. 4 Microbiome promotes aggression in female flies. (**a**) The number of head butting in GF females was significantly lower than that in CR and MB females. (**b**) Latency to the first head butting in GF females was significantly longer than that in CR and MB females. $n = 15$ for each. For all variables have different letters, they are significantly different ($p < 0.05$). Kruskal-Wallis test followed by Dunn's multiple comparisons test.

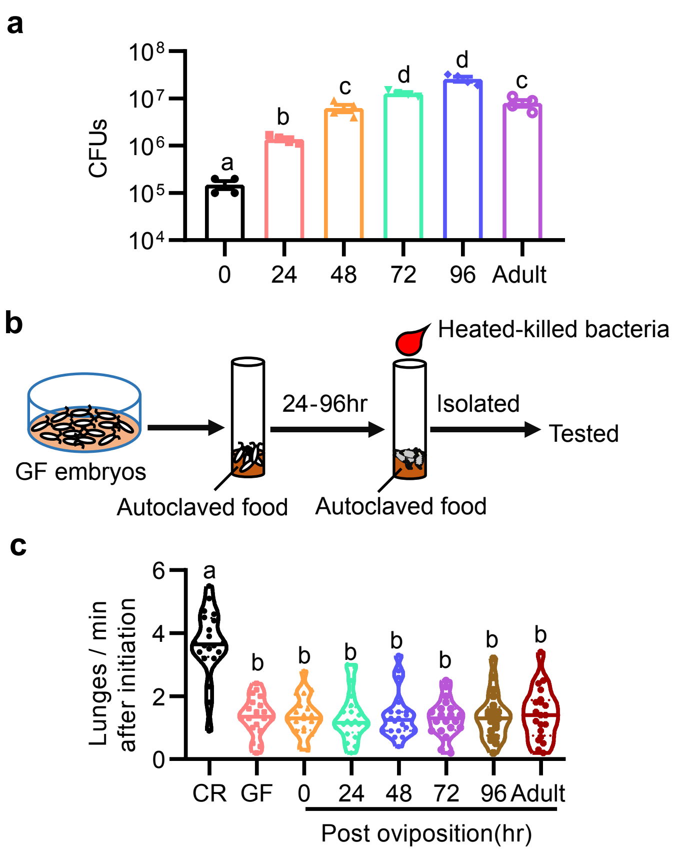

Supplementary Fig. 5 Association with high titres of heat-killed bacteria does not enhance male aggression. (**a**) Number of colony-forming units (CFUs) from fly food cultured with CR flies over time. $n = 4$ for each. Error bars indicate SEM. (**b**) Experimental setup to assess the impact of dead bacteria in massive excess on male aggression. (**c**) Lunging frequencies of GF males associated with 10-fold amounts of heat-killed bacteria (~2.6 x 10^8 CFU) at specific developmental periods were not significantly different from GF males. $n = 20$ for each. For all variables have different letters, they are significantly different ($p < 0.05$). Kruskal-Wallis test followed by Dunn's multiple comparisons test.

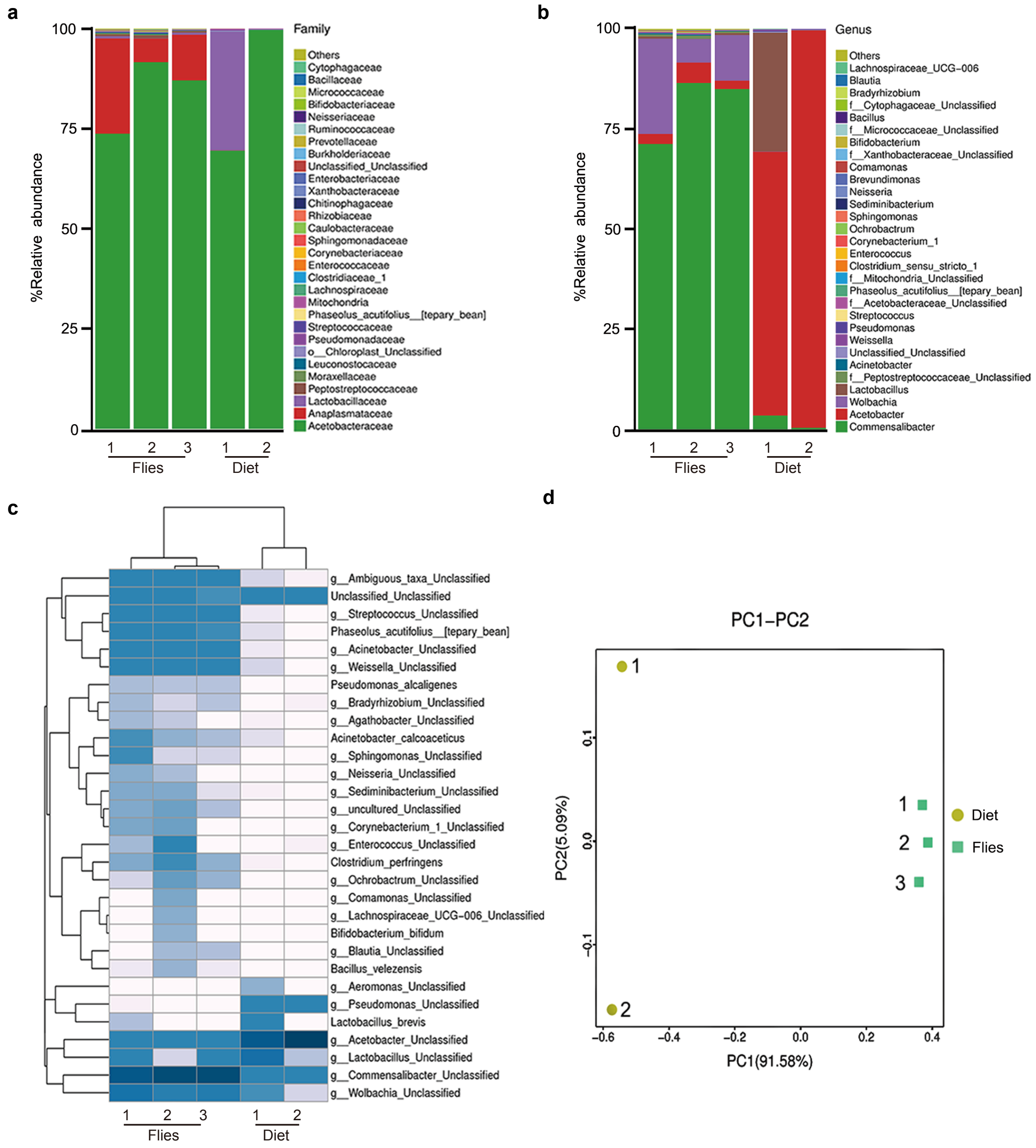

Supplementary Fig. 6 The composition of microbiome in fly food and *Drosophila* guts. (**a** and **b**) Composition and distribution of the dominant bacterial taxa within food and *Drosophila* at the family (**a**) and genus level (**b**). (**c**) Hierarchical clustering diagram using the average-neighbor (HC-AN) method comparing samples of fly diet (n = 2) and intestinal microbiome (n = 3). CR

flies and regular fly food were used. Associated heatmap shows the relative abundance of representative. One representative OTU with the greatest difference between the two group means from each family is selected for inclusion in the heatmap diagram. OTUs are shown as: Phylum, Class, Order, Family, Genus, and Species. (**d**) Principal coordinates analysis (PCA) of unweighted jack-knifed UniFrac distances of bacterial communities for diet and fly guts. PC1, principal coordinate 1; PC2, principal coordinate 2.

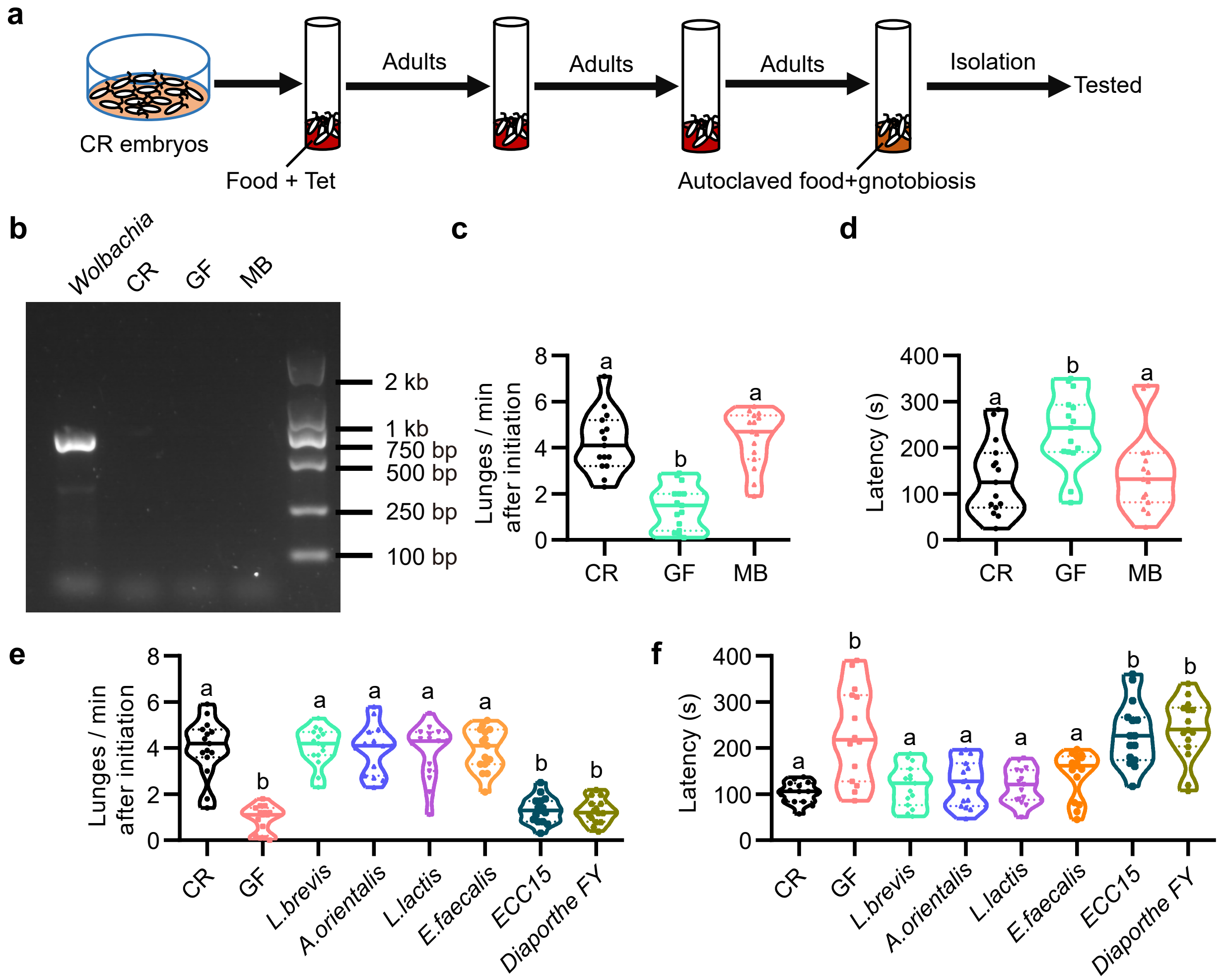

Supplementary Fig. 7 Effect of different microbial species on *Drosophila* male aggression. (a) A diagram to generate *Wolbachia*-free fly lines by raising them on standard fly food with 0.05

mg/ml of hydrochloride tetracycline for successively three generations. *Wolbachia*-free flies were used for aggression assay. (**b**) The presence/absence of *Wolbachia* infection in flies was examined using a PCR-based assay with *Wolbachia*-specific primers. Experiments were repeated for 3 times. (**c** and **d**) Lunging frequency (**c**) and latency (**d**) of *Wolbachia*-free CR, GF and MB pairs. $n = 15$ for each. (**e** and **f**) Lunging frequency (**e**) and latency (**f**) in males that were associated with commensal and pathogenic microbes. $n = 15$ for each. For all variables have different letters, they are significantly different ($p < 0.05$). One-way ANOVA followed by Tukey's multiple comparisons test.

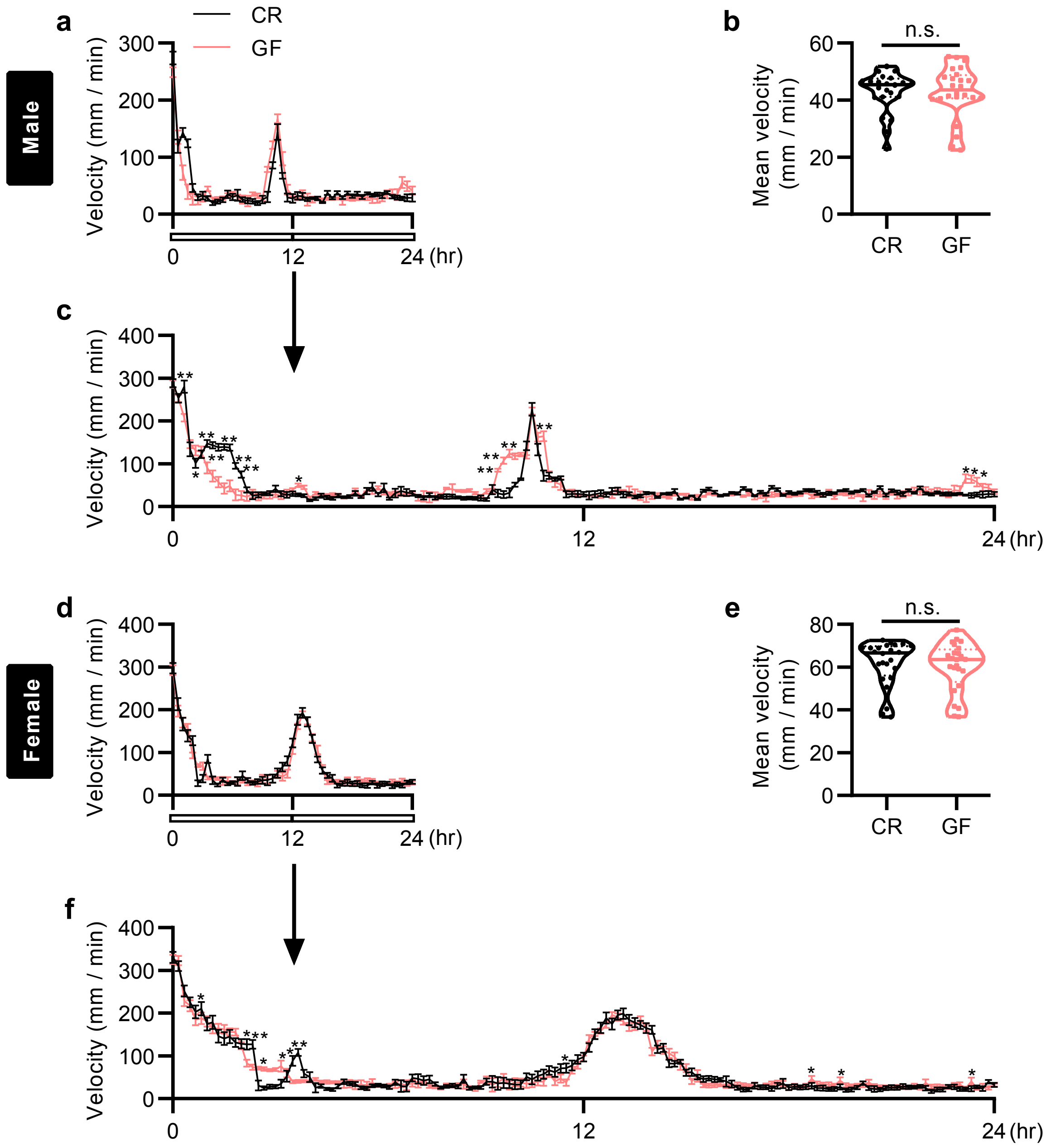

Supplementary Fig. 8 Locomotor behaviors are not affected in GF flies generated using another method. (**a** and **b**) 24-hr walking speeds (**a**) and the average velocity (**b**) of CR and GF males were not significantly different. n = 24 for each. (**c**) The average velocity of CR and GF males every 10 min for 24 hr. n = 24 for each. (**d** and **e**) 24-hr walking speeds (**d**) and the average velocity (**e**) of CR and GF females were not significantly different. n = 24 for each. (**f**) The average velocity of

CR and GF females every 10 min for 24 hr. *$p < 0.05$, **$p < 0.01$, $n = 24$ for each. n.s., not significant, Two-tailed unpaired Student's t-test (**b**, **c**, **e** and **f**). GF flies were generated using a protocol described in Supplementary Fig. 2d. Error bars indicate SEM.

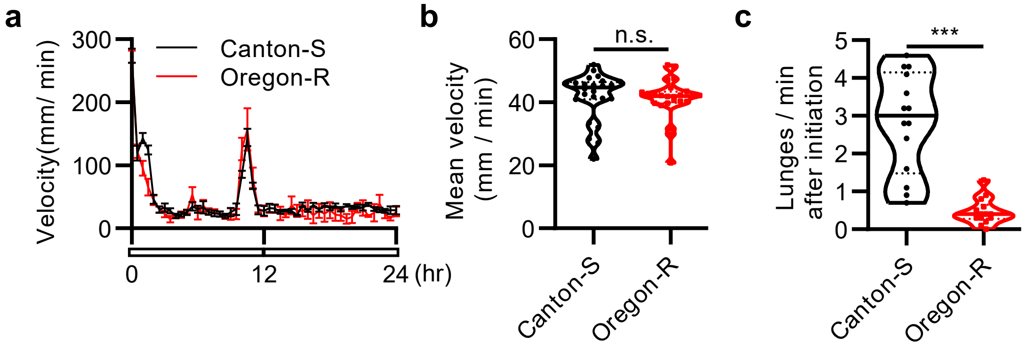

Supplementary Fig. 9 Locomotor and aggressive behaviors in wild type Canton-S and Oregon-R flies. (**a** and **b**) 24-hr walking speeds (**a**) and the average velocity (**b**) of Canton-S and Oregon-R males were not significantly different. $n = 24$ for each. n.s., not significant, Mann-Whitney U test. Error bars indicate SEM. (**c**) Comparison of intermale aggression of the two wild-type strains. $n = 14$ for each. ***$p < 0.001$, Two-tailed unpaired Student's t-test.

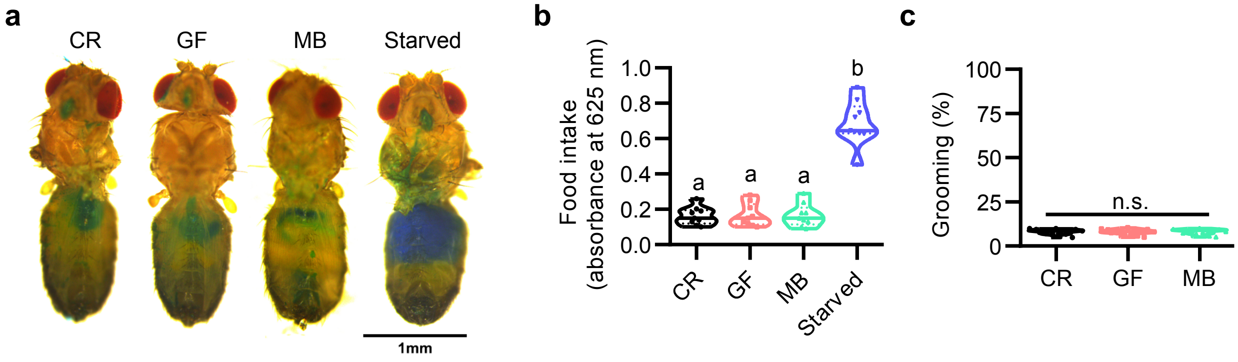

Supplementary Fig. 10 Decreased aggression in GF males was not due to potential elevation of feeding and grooming behaviors. (**a**) Dye-stained food in the abdomen of fed CR, GF and MB

males, and starved CR males as a positive control, under a 30-min feeding assay. Fed CR, GF and MB males were used for feeding assay to mimic the feeding condition in the aggression assay. (**b**) Colorimetric quantification of food intake in CR, GF and MB males. Food consumption was evaluated with spectroscopy-based quantification of blue-dye levels. $n = 9$ for each. (**c**) Grooming is equally low in CR, GF and MB males during aggression assay. $n = 15$ for each. For all variables have different letters, they are significantly different ($p < 0.05$). Significance was tested using a Kruskal–Wallis test with Dunn's multiple comparison test.

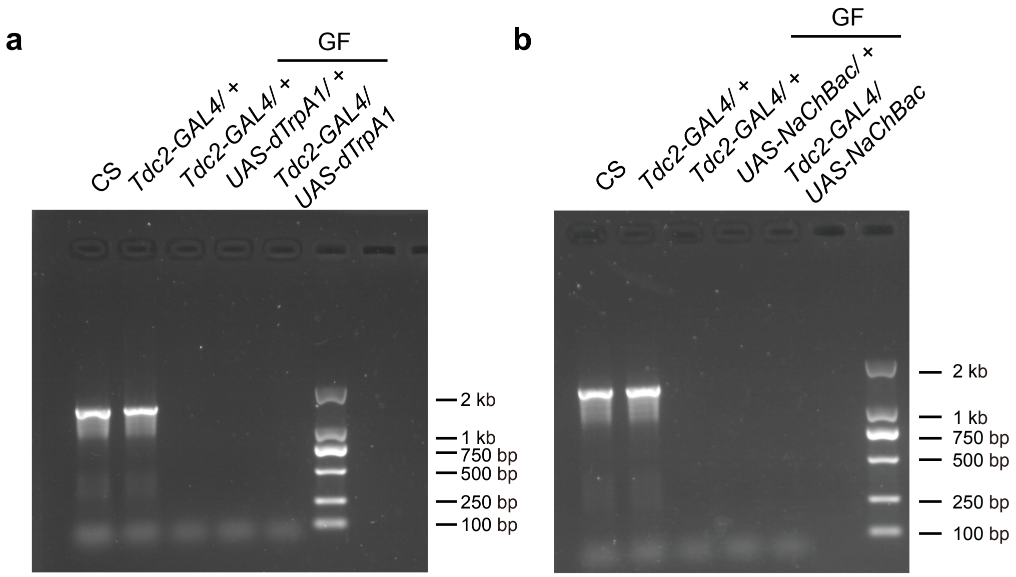

Supplementary Fig. 11 Verification of axenia in GF flies. (**a**) Representative images of DNA agarose electrophoresis with16S rDNA PCR samples to examine axenia in GF flies expressing dTrpA1 in Tdc2 neurons. (**b**) Representative images of DNA agarose electrophoresis using 16S rDNA PCR samples to examine axenia in GF flies expressing NaChBac in Tdc2 neurons. Experiments were repeated for 3 times. Conventionally reared CS and *Tdc2-GAL4/+* were used as positive controls.

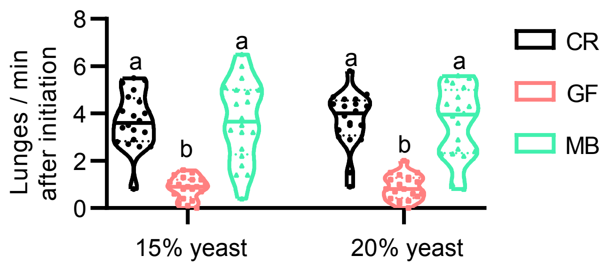

Supplementary Fig. 12 Excessive nutrition fails to promote aggression in GF flies. GF flies raised on autoclaved fly diet with 15% or 20% yeast exhibited a significant decrease in lunge behavior compared to that of CR and MB flies. $n = 15$ for each. For all variables have different letters, they are significantly different ($p < 0.05$). Kruskal-Wallis test followed by Dunn's multiple comparisons test.

Supplementary Table 1. Primer sequences used in this study.

Gene	Sequence	
Tdc2	F: CTCGCTTCCACGCCTATT	R: GTCGTCGGGCTCCAGAAT
Tβh	F: GCGATGAGATGTGCGTCAAC	R: ACAGCGGATTGTAGTTGGGG
TH	F: GCAGACCAAACAAACCGTCC	R: AATCCGGGGTGGTTCATGTC
Trh	F: GCTGGCCAACTCCAGTTTTG	R: ATTTGCTCCTTGGCCTCCTC
Tk	F: CATCCATGGCTCTACCTGTG	R: GCTCTAAGAGGAACTTCCATC
Gad1	F: GAATTCAGAGCATAGAAGCCACCG	R: CAACTGGCTCTTCATCTTCTCCTG
