# Supplementary material for: Gut microbiome modulates Drosophila aggression through octopamine signaling
Source: Nat Commun. 2021 May 11;12:2698. doi: 10.1038/s41467-021-23041-y (PMC8113466; doi:10.1038/s41467-021-23041-y)
Supplement: Supplementary file 3 — Description of Additional Supplementary Files [file 41467_2021_23041_MOESM3_ESM.pdf]

Description of Additional Supplementary Files

Supplementary Movie 1. Aggression assay between two CR males.

Supplementary Movie 2. Aggression assay between two GF males.

Supplementary Movie 3. Aggression assay between a CR and a GF male. The CR male was dotted with blue on the thorax, and the GF male was dotted with red.

Supplementary Movie 4. A GF male is less competitive for mating with a female compared with a MB male. The GF male was dotted with red on the thorax, and the MB male was dotted with blue.
