# Supplementary material for: Gut microbiome modulates Drosophila aggression through octopamine signaling
Source: Nat Commun. 2021 May 11;12:2698. doi: 10.1038/s41467-021-23041-y (PMC8113466; doi:10.1038/s41467-021-23041-y)
Supplement: Supplementary file 8 — Reporting summary [file 41467_2021_23041_MOESM8_ESM.pdf]

nature research

Corresponding author(s): Wei Liu & Yufeng Pan

Last updated by author(s): Mar 29, 2021

Reporting Summary

Nature Research wishes to improve the reproducibility of the work that we publish. This form provides structure for consistency and transparency in reporting. For further information on Nature Research policies, see Authors & Referees and the Editorial Policy Checklist .

Statistics

For all statistical analyses, confirm that the following items are present in the figure legend, table legend, main text, or Methods section.

n/a	Confirmed	
☐	☒	The exact sample size (*n*) for each experimental group/condition, given as a discrete number and unit of measurement
☐	☒	A statement on whether measurements were taken from distinct samples or whether the same sample was measured repeatedly
☐	☒	The statistical test(s) used AND whether they are one- or two-sided<br>*Only common tests should be described solely by name; describe more complex techniques in the Methods section.*
☒	☐	A description of all covariates tested
☐	☒	A description of any assumptions or corrections, such as tests of normality and adjustment for multiple comparisons
☐	☒	A full description of the statistical parameters including central tendency (e.g. means) or other basic estimates (e.g. regression coefficient) AND variation (e.g. standard deviation) or associated estimates of uncertainty (e.g. confidence intervals)
☐	☒	For null hypothesis testing, the test statistic (e.g. *F*, *t*, *r*) with confidence intervals, effect sizes, degrees of freedom and *P* value noted<br>*Give P values as exact values whenever suitable.*
☒	☐	For Bayesian analysis, information on the choice of priors and Markov chain Monte Carlo settings
☒	☐	For hierarchical and complex designs, identification of the appropriate level for tests and full reporting of outcomes
☒	☐	Estimates of effect sizes (e.g. Cohen's *d*, Pearson's *r*), indicating how they were calculated

Our web collection on statistics for biologists contains articles on many of the points above.

Software and code

Policy information about availability of computer code

Data collection	Video recording: Sony FDR-AX40; sleep: DAM System 308; qPCR: Light Cycler 96; locomotion: ZebraLab software system; Confocal data: Zeiss 700 or 710; HPLC/MS analysis: Agilent MSD/QTOF 6545 system and HPLC/1290II; 16S rRNA sequencing and analysis: QIIME software with Python scripts.
Data analysis	Courtship: LifeSong X; sleep: DAMFile Scan113 and Matlab-R2018a; locomotion: ZebraLab software system; confocal images: ImageJ 1.52v; Figure drawing: Prism 8 and Adobe Illustrator CC 2019; Statistics: Prism 8.

For manuscripts utilizing custom algorithms or software that are central to the research but not yet described in published literature, software must be made available to editors/reviewers. We strongly encourage code deposition in a community repository (e.g. GitHub). See the Nature Research guidelines for submitting code & software for further information.

Data

Policy information about availability of data

All manuscripts must include a data availability statement. This statement should provide the following information, where applicable:

- Accession codes, unique identifiers, or web links for publicly available datasets
- A list of figures that have associated raw data
- A description of any restrictions on data availability

All data generated or analyzed during this study are included in the manuscript and its supplementary information files. All other relevant data supporting the findings of this study are available from the corresponding author upon reasonable request. Source data are provided with this paper.

Field-specific reporting

Please select the one below that is the best fit for your research. If you are not sure, read the appropriate sections before making your selection.

- [x] Life sciences
- [ ] Behavioural & social sciences
- [ ] Ecological, evolutionary & environmental sciences

For a reference copy of the document with all sections, see nature.com/documents/nr-reporting-summary-flat.pdf

Life sciences study design

All studies must disclose on these points even when the disclosure is negative.

Sample size	Sample size for male courtship, aggression, sleep or locomotion test is normally around 20 for each group, for tissue staining and quantification ≥6. All samples sizes are indicated in each figure legend. Sample sized were predetermined based on previous studies in this field (about 20 for behavioral experiments and 5-10 for confocal experiments).
Data exclusions	No data were excluded from the analyses.
Replication	All data presented are representative of at least two independent experiments as indicated in the method part of statistics, and replications were successful.
Randomization	Randomization was irrelevant for the design of this study. All the experimental groups were allocated based on genotypes, and appropriate control groups were assayed in parallel.
Blinding	Investigators were not blinded during data collection and analysis. Experimental manipulation due to implicit bias was not possible without actively tampering with the raw data which are mostly videos and confocal images.

Reporting for specific materials, systems and methods

We require information from authors about some types of materials, experimental systems and methods used in many studies. Here, indicate whether each material, system or method listed is relevant to your study. If you are not sure if a list item applies to your research, read the appropriate section before selecting a response.

Materials & experimental systems

n/a	Involved in the study	
[ ]	[x]	Antibodies
[x]	[ ]	Eukaryotic cell lines
[x]	[ ]	Palaeontology
[ ]	[x]	Animals and other organisms
[x]	[ ]	Human research participants
[x]	[ ]	Clinical data

Methods

n/a	Involved in the study	
[x]	[ ]	ChIP-seq
[x]	[ ]	Flow cytometry
[x]	[ ]	MRI-based neuroimaging

Antibodies

Antibodies used	Primary antibodies include rabbit anti-Tdc2 (pab0822-P, covalab, France) 1:400, mouse anti-Bruchpilot (nc82, Developmental Studies Hybridoma Bank) 1:50, and secondary Alexa Fluor 488 (1:500, A-11029, Invitrogen) and 568 antibodies (1:500, A-11004, Invitrogen).
Validation	The validation statement of Tdc2 antibody can be found on the manufacturer's website(https://www.covalab.com/tyrosine-decarboxylase-2-antibody-3.htm). Anti-Bruchpilot, secondary Alexa Fluor 488 and 568 antibodies are validated by the supplier by either immunoprecipitation by a cell lysate and analyzed by mass spectrometry, western blot and detecting the protein in tissue or cells. All the above antibodies were commonly used by the field.

Animals and other organisms

Policy information about studies involving animals; ARRIVE guidelines recommended for reporting animal research

Laboratory animals	We used Drosophila melanogaster in this study. Wild-type Canton-S were used as wild-type throughout except for Supplementary Fig. 9 which also used Oregon-R.<br>For aggression, feeding, HPLC/MS or qRT-PCR experiments, 5-7 days old males were used in Figure 1d-i, Figure 3c, Figure 4a, c, d, h-j, Figure 5b-f, Figure 6b-g, Supplementary Fig. 2, Supplementary Fig. 3, Supplementary Fig. 5c, Supplementary Fig. 7, Supplementary Fig. 9c, Supplementary Fig. 10 and Supplementary Fig. 12.<br>5-7 days old females were used in Figure 4b and Supplementary Fig. 4 for qRT-PCR or aggression assays.<br>For courtship assays, 4-6 days old males and 5-7 days old virgin females were used in Figure 3a, b and d.

	For locomotion assays, 4-6 days old males were used in Figure 2a, b and Supplementary Fig. 8a-c, and 4-6 days old females were used as in Figure 2e, f and Supplementary Fig. 8d-f.<br>For sleep test, 3-5 days old male were used in Figure 2c and d, and 3-5 days old females were used in Figure 2g and h.<br>For confocal experiments, 4-6 days old males were used in Figure 4e-g, and L2, L3 larva were used in Figure 5g-l.
Wild animals	The study did not involve wild animals.
Field-collected samples	The study did not involve samples collected from the field.
Ethics oversight	No ethical oversight was required as no vertebrate animals were involved in the research, and no ethical oversight of the experiments was required by our institution.

Note that full information on the approval of the study protocol must also be provided in the manuscript.
